# Recent trends of incidence and mortality of cutaneous lymphomas in Germany

**DOI:** 10.1111/ddg.15904

**Published:** 2025-12-12

**Authors:** Khodr Cheikh El Najjarine, Hiltraud Kajüter, Ina Wellmann, Andreas Stang, Chalid Assaf

**Affiliations:** ^1^ Department of Dermatology HELIOS Klinikum Krefeld Academic Teaching Hospital of the University of Aachen Krefeld Germany; ^2^ Cancer Registry of North Rhine‐Westphalia Bochum Germany; ^3^ Institute for Medical Informatics Biometry and Epidemiology University Hospital Essen University of Duisburg‐Essen Essen Germany; ^4^ Institute for Molecular Medicine Medical School Hamburg Hamburg Germany

**Keywords:** Cutaneous lymphomas, CTCL, CBCL, epidemiology, incidence, mortality, registry, survival

## Abstract

**Background:**

Primary cutaneous lymphomas (PCL) are primarily composed of cutaneous T‐cell lymphomas (CTCL), followed by cutaneous B‐cell lymphomas (CBCL). This study aimed to assess the incidence and survival rates of PCL in Germany.

**Methods:**

We analyzed data from the North Rhine‐Westphalia Cancer Registry (2008–2021), which covers a population of 18 million. Age‐standardized incidence rates and relative survival were calculated.

**Results:**

The analysis included 3,853 patients with newly diagnosed PCL. Of these, 69.5% were CTCL, and 24.9% were CBCL. The age‐standardized incidence of PCL was 10.8 per million person‐years. The incidence of both CTCL and CBCL increased over time. PCL cases were also identified in children. The overall five‐year relative survival for PCL was 89% (95% CI: 87–92%), with CTCL patients having a survival rate of 91% (95% CI: 88–94%) and CBCL patients of 86% (95% CI: 81–91%). Among patients with Sézary syndrome, the five‐year relative survival was 53% (95%: CI:29–77%).

**Conclusions:**

This study represents the largest population‐based analysis of PCL in Germany, including both adults and children. The incidence of PCL was higher than previously reported. Additionally, we present the first survival data for PCL in Germany, revealing a notably higher survival probability for patients with Sézary syndrome.

## INTRODUCTION

Primary cutaneous lymphomas (PCL) are a heterogeneous group of skin disorders that vary widely in symptoms and prognosis.^1^, ^2^ They are the second most common type of extranodal non‐Hodgkin lymphoma with primary skin involvement, following gastrointestinal lymphomas.^1^, ^3^, ^4^ PCLs are defined by the presence of skin‐only manifestations at the time of diagnosis.

The global incidence of PCL is estimated at approximately 1 per 100,000 individuals annually. Among these, 73% are cutaneous T‐cell lymphomas (CTCL), 22% are cutaneous B‐cell lymphomas (CBCL), and the remainder are rare subtypes originating from natural killer cells or plasmacytoid dendritic cells.^1^, ^2^, ^3^, ^4^ Each subtype of PCL has a distinct prognosis, ranging from indolent, long‐term courses to aggressive disease with poor survival outcomes.^1^, ^5^, ^6^, ^7^

Epidemiological data on primary cutaneous lymphomas (PCL) from population‐based studies have historically been limited due to the disease's rarity and heterogeneity. Additionally, the classification of PCL has evolved over the past 50 years, complicating comparisons between historical and current data. Since the introduction of the WHO–EORTC classification system in 2005, which incorporates clinical, histological, and molecular genetic features, the classification of PCL has become more consistent and globally comparable.^1^

Recent studies have revealed regional differences in the incidence and subtype distribution of PCL.^6^ For example, rare subtypes such as EBV‐associated NK/T‐cell lymphomas are more prevalent in Asian countries, while other subtypes are more evenly distributed worldwide. Dobos et al. reported an increased incidence of PCL in the French Cutaneous Lymphomas Registry between 2005 and 2019.^8^ In the Netherlands, Ottenvanger et al. observed a significant rise in mycosis fungoides and Sézary syndrome cases between 2000 and 2020.^7^

In Greece, Kaliampou et al. analyzed PCL incidence and subtypes at Attica's main hematopathology center from 2009 to 2021.^8^ Their findings suggest a higher national incidence of 2.2 new cases per 100,000 individuals (European Standard Population) – potentially due to improved diagnostic capabilities. Similarly, Titou et al. conducted a retrospective analysis of 114 mycosis fungoides cases (1993–2022), finding a higher prevalence among older males, with overall survival of 85.7% at 5 years, 74.6% at 10 years, and 61.4% at 20 years.^9^

Regional differences in age distribution have also been noted. A study from Saudi Arabia reported a median age of 41 years among CTCL patients, linked to a notably high pediatric incidence of 12.8%.^10^ This rate is even higher in the Far East, where up to 25% of CTCL cases occur in children.^11^

The most recent large‐scale registry‐based epidemiological data on PCL in Germany were published by Assaf et al. in 2007, based on a registry involving 998 patients from 26 dermatology departments.^12^ This study provided foundational data on patient demographics and subtype distribution.

In the present study, we offer a comprehensive analysis of PCL incidence and relative survival – including subtype‐specific outcomes – for the first time in Germany. The data are drawn from the North Rhine‐Westphalian cancer registry, which serves a population of 18 million and includes reported cutaneous lymphoma cases from 2008 to 2021.

## MATERIAL AND METHODS

The Cancer Registry of North Rhine‐Westphalia (LKR NRW) covers a population of approximately 18 million people, representing about 22% of Germany's total population. Cancer reporting is mandatory for all diagnosing and treating physicians, including pathologists and dermatopathologists. The completeness of the registry is routinely assessed by the *Center for Cancer Registry Data* at the Robert Koch Institute and exceeds 95% for the LKR NRW.

Primary cutaneous lymphomas (PCL) cases were identified using ICD‐O morphology codes 9590/3–9729/3 (Hodgkin and Non‐Hodgkin lymphomas) and ICD‐O topography codes C44 (skin), C51 (vulva), C60 (penis), or C63.2 (scrotum), based on the International Classification of Diseases for Oncology, 3rd Edition (ICD‐O‐3).

The analysis was further restricted to cases coded C82–C88 according to the 10th Revision of the International Classification of Diseases (ICD‐10). The dataset includes PCL cases diagnosed between January 2008 and December 2021.

We analyzed PCL overall, as well as cutaneous T‐cell lymphomas (CTCL) and cutaneous B‐cell lymphomas (CBCL) separately. These groups were further stratified by ICD‐10 codes. Among CTCL, the WHO–EORTC^1^ classification distinguishes mycosis fungoides (MF, C84.0), Sézary syndrome (SS, C84.1), and primary cutaneous CD30‐positive T‐cell proliferations (pcCD30^+^LPD, C86.6). For CBCL, it includes primary cutaneous follicular lymphoma (pcFL, C82), primary cutaneous diffuse large B‐cell lymphoma (pcDLBCL, C83.3), and primary cutaneous marginal zone lymphoma (pcMZL, C88.4).

The following variables were considered in the analysis: sex, age at diagnosis, histopathology, entity, anatomic location and date of death.

### Statistical methods

Age‐standardized incidence rates for the study period January 2008 to December 2021 were calculated using the “old” European Standard Population.^13^ Cases in which the death certificate was the only source of information (Death Certificate Only, DCO) were included in incidence calculations but excluded from survival analyses due to the lack of detailed clinical data.

We estimated five‐year relative survival using the period analysis approach, based on survival experiences during the calendar period 2017–2021.^14^, ^15^ This method incorporates not only patients diagnosed between 2017 and 2021, but also those diagnosed in the preceding 5 years who survived into the 2017–2021 interval, thus providing more up‐to‐date survival estimates.

To assess the precision of our estimates, we calculated and reported standard errors (SE) and confidence intervals (CI). As the primary goal of this study is estimation rather than hypothesis testing, we avoid emphasizing statistical significance and instead focus on the precision and validity of our results to reduce the risk of publication bias.^16^, ^17^

All analyses were conducted using SAS software version 9.4 (SAS Institute, Cary, NC).

## RESULTS

### Incidence and demographics

From 2008 to 2021, a total of 3,853 primary cutaneous lymphoma (PCL) cases were reported to the Cancer Registry of North Rhine‐Westphalia by clinicians, pathologists, and dermatopathologists. Among these, 2,680 cases (69.6%) were CTCL, 960 cases (24.9%) were CBCL, and 213 cases (5.5%) represented unspecified or rare subtypes (Table 1).

**TABLE 1:** Number and age‐standardized incidence rates of recorded incident cutaneous lymphoma cases in the population‐based cancer registry of North Rhine‐Westphalia, Germany, 2008–2021.

	Total				Men				Women			
*Types of cutaneous lymphoma*	*n*	*%*	*ASR*	*SE*	*n*	*%*	*ASR*	*SE*	*n*	*%*	*ASR*	*SE*
Overall	3.853		10.8	0.18	2386		14.2	0.30	1.467		7.7	0.22
***Cutaneous T‐cell lymphoma***	2.680	100	7.5	0.15	1.756	100	10.4	0.26	924	100	5.1	0.18
Mycosis fungoides (C84.0)	1.827	68.2	5.1	0.13	1.246	71.0	7.4	0.22	581	62.9	3.2	0.14
Sézary syndrome (C84.1)	94	3.5	0.2	0.02	45	2.6	0.2	0.03	49	5.3	0.2	0.03
Primary cutaneous CD30‐positive T‐cell proliferations (C86.6)	91	3.4	0.3	0.03	49	2.8	0.3	0.04	42	4.5	0.3	0.04
Other T‐cell‐lymphomas	668	24.9	1.9	0.08	416	23.7	2.5	0.13	252	27.3	1.4	0.10
***Cutaneous B‐cell lymphoma***	960	100	2.7	0.09	516	100	3.2	0.15	444	100	2.2	0.11
Follicular lymphoma (C82)	370	38.5	1.1	0.06	209	40.5	1.3	0.10	161	36.3	0.9	0.07
Diffuse large B‐cell lymphoma (C83.3)	254	26.5	0.6	0.04	125	24.2	0.7	0.06	129	29.1	0.4	0.04
Marginal zone lymphoma (C88.4)	320	33.3	1.0	0.06	171	33.1	1.1	0.09	149	33.6	0.8	0.07
Other B‐cell‐lymphomas	16	1.7	0.0	0.01	11	2.1	0.1	0.02	5	1.1	0.0	0.01
***Other & unspecified lymphoma***	213		0.6	0.04	114		0.7	0.06	99		0.5	0.05
***Age of diagnosis: Median (IQR)***	68.8 (57.4, 78.4)					68.7 (57.3, 77.9)				69.0 (57.4, 79,3)		

*Abbr*.: ASR, age‐standardized rate; SE, standard error; IQR, interquartile range

All rates are expressed as cases per million person‐years; rates are standardized to the old European standard population.

**FIGURE 1:**
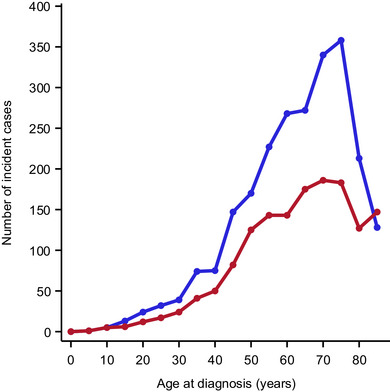
Age distribution of male and female incident cases of cutaneous lymphoma in North Rhine‐Westphalia, Germany, 2008–2021. Males, blue graph; females, red graph. The median age at diagnosis and the 25th and 75th percentile was 67.3 years (55.3, 76.3) among men and 67.6 years (55.1, 77.3) among women.

The age‐standardized incidence rate of PCL was 14.2 per million person‐years in men and 7.7 in women, with an overall average incidence of 10.8 per million person‐years. Incidence rates rose exponentially with age, peaking in individuals aged 60 years and older. The median age at diagnosis was 66.9 years (67.6 years for men, 65.7 years for women). Notably, 0.8% of cases occurred in individuals under 20 years of age (Figure 1).

Among men, the age‐specific incidence rates for CTCL and CBCL diverged significantly with age, particularly peaking in the 70–74 age group. In contrast, younger women (ages 25–49) showed higher incidence rates of CTCL compared to CBCL, though this gap narrowed by age 55–59. For CTCL, the sex difference in incidence widened with age, a pattern not observed for CBCL (online supplementary Figures S1 and S2).

Overall, 2,386 men (61.9%) and 1,467 women (38.1%) were diagnosed with PCL, yielding a male‐to‐female ratio of 1.6:1. Most PCL subtypes were more frequent in males. Among CTCL, mycosis fungoides was the most common subtype (Table 1). For CBCL, the indolent subtypes – pcFL and pcMZL – were most prevalent, followed by the aggressive pcDLBCL (Table 1).

### Temporal trends in incidence

**FIGURE 2:**
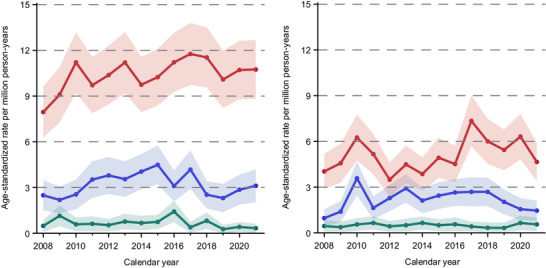
Age‐standardized incidence rates of cutaneous lymphoma in North Rhine‐Westphalia, Germany, 2008–2021, by sex. Red graphs, CTCL (cutaneous T‐cell lymphoma); blue graphs, CBCL (cutaneous B‐cell lymphoma); green graphs, other cutaneous lymphoma.

From 2008 to 2021, the age‐standardized incidence rates for both CTCL and CBCL increased among both, men and women (CTCL men: from 7.95 to 10.74 per 100,000 person‐years [+35.1%], women: from 4.03 to 4.65 per 100,000 person‐years [+15.5%]; CBCL men: from 2.49 to 3.11 per 100,000 person‐years [+25.0%], women: from 0.96 to 1.46 per 100,000 person‐years [+5.3%]). In terms of the absolute case counts, the number of CTCL cases increased from 93 to 123 (men) and 54 to 65 (women), and the number of CBCL cases increased from 25 to 35 cases (men) and 12 to 26 cases (women) per year (Figure 2).

### Survival Analysis

Five‐year relative survival was estimated for the period 2017–2021 (Table 2). The overall five‐year relative survival for PCL was 89.0%, with survival higher in men (90.8%) than in women (86.1%).

**TABLE 2:** Five‐year relative survival estimates (%) for 2017–2021 in patients with newly diagnosed cutaneous lymphomas in North Rhine‐Westphalia, Germany.

	Total		
*Types of cutaneous lymphomas*	*n*	*Estimate*	*95% Cl*
Overall	2,674	89.0	(86.5; 91.5)
Overall, men	1,656	90.8	(87.6; 94)
Overall, women	991	86.1	(82.1; 90.1)
***Cutaneous T‐cell lymphomas***	1,835	91.0	(88.1; 93.9)
Mycosis fungoides (C84.0)	1,266	96.2	(93; 99.4)
Sézary syndrome (C84.1)	42	53.0	(28.9; 77.2)
Primary cutaneous CD30‐positive T‐cell proliferations (C86.6)	66	98.2	(85.6; 110.7)
Other T‐cell‐lymphomas	461	80.2	(74; 86.3)
***Cutaneous B‐cell lymphomas***	677	86.0	(80.7; 91.2)
Follicular lymphomas (C82)	288	100.6	(95.2; 106)
Diffuse large B‐cell lymphomas (C83.3)	146	55.5	(42.3; 68.7)
Marginal zone lymphomas (C88.4)	233	92.3	(84.1; 100.4)
Other B‐cell‐lymphomas	10	–	–
***Other & unspecified lymphomas***	135	80.0	(68; 92)

*Abbr*.: CI, confidence interval.

**FIGURE 3:**
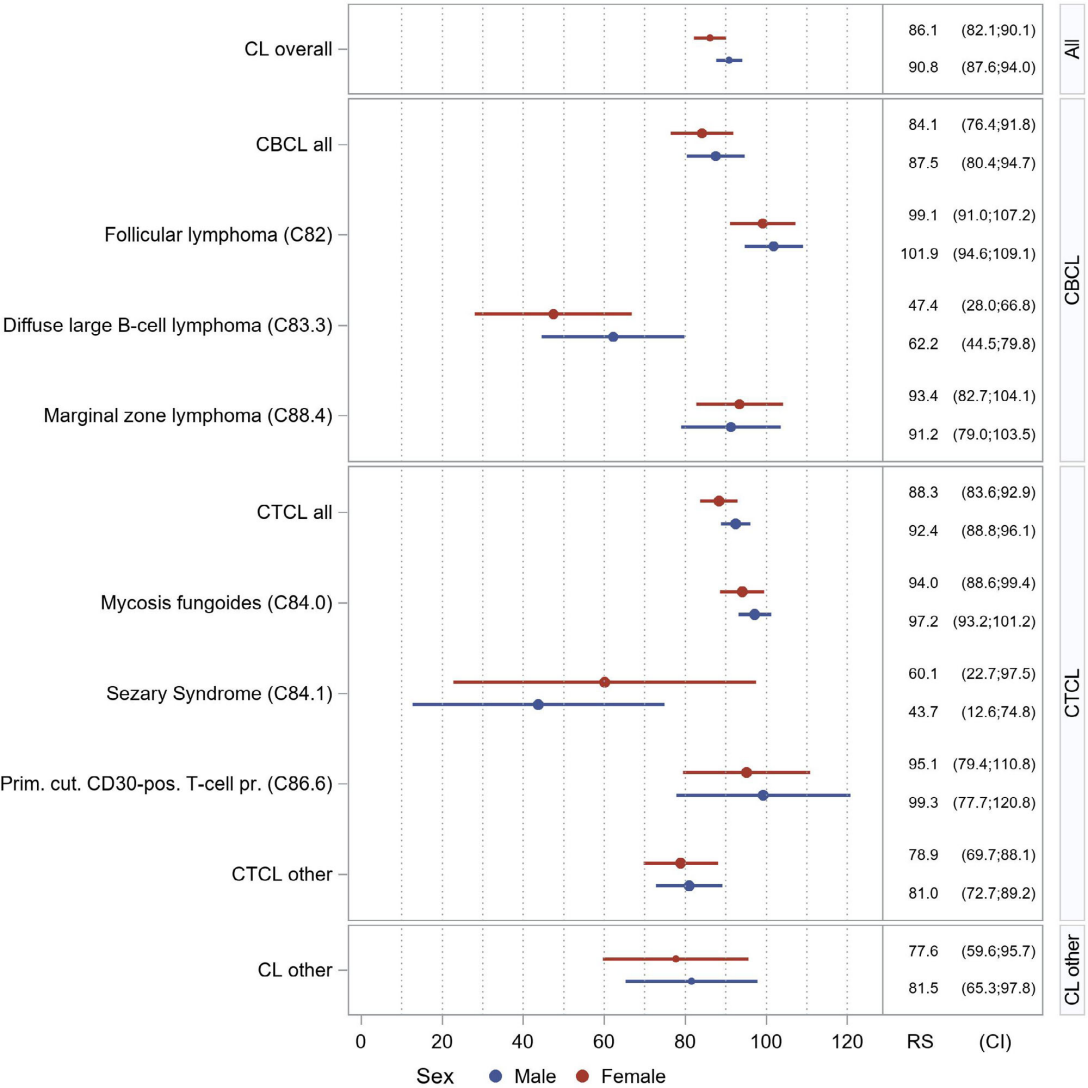
Relative survival, 2017–2021. CL, cutaneous lymphoma; CTCL, cutaneous T‐cell lymphoma; CBCL, cutaneous B‐cell lymphoma; C82, follicular lymphoma; C83.3, diffuse large B‐cell lymphoma; C88.4, marginal zone lymphoma; C84.0, mycosis fungoides; C84.1, Sézary syndrome; C86.6, primary cutaneous CD30‐positive T‐cell proliferations.

Survival by PCL subtypes showed CTCL overall: 91.0%, MF: 96.2%, SS: 53.0%, pcCD30+LPD: 98.2%, unspecified CTCL: 90.2%. CBCL overall: 86.0%, pcFL: 100%, pcDLBCL: 55.5%, pcMZL: 92.3%. Due to the limited number of unspecified CBCL cases, five‐year survival could not be reliably estimated for this group (Table 2 and Figure 3).

## DISCUSSION

We present population‐based incidence and survival data for primary cutaneous lymphomas (PCL) from 2008 to 2021, derived from the Cancer Registry of North Rhine‐Westphalia (LKR NRW) – the largest cancer registry in Germany, covering approximately 18 million inhabitants. This dataset allows for an in‐depth analysis of epidemiological patterns and temporal changes in both CTCL and CBCL.

The age‐standardized incidence rate for PCL in North Rhine‐Westphalia was 1.08 per 100,000 person‐years, higher than reported in France (0.96), the U.S. (0.87), and consistent with other European registries, though still lower than in Greece (2.2).^6^, ^8^, ^18^ This underscores regional variability in incidence, possibly reflecting differences in diagnostic practices, registry completeness, or underlying population risk factors.

In our cohort, CTCL constituted 69.9% of all PCL cases, which is consistent with prior findings (70–85%).^6^, ^12^, ^19^ The CTCL incidence rate in our data (7.5 per million person‐years) was notably higher than rates reported in the SEER Program of 1.4 per million,^20^, ^21^, ^22^, ^23^, ^24^ (Surveillance, Epidemiology, and End Results, a population‐based cancer registry program maintained by the National Cancer Institute in the United States) and other European datasets.^6^, ^12^ The predominance of MF among CTCL subtypes (68.2%) aligns with European trends,^8^, ^10^, ^15^ but exceeds the U.S. SEER‐reported proportion of 56.6%.^24^

This discrepancy may reflect differences in classification accuracy. SEER data show a larger proportion of CTCL cases categorized as “other,” possibly due to incomplete diagnostic information. In contrast, our registry, based heavily on pathology reports, may overrepresent histologically confirmed entities like peripheral T‐cell lymphoma (PTCL), while underrepresenting diagnoses like CD30^+^LPD, which rely on clinical context.

Furthermore, SEER data have demonstrated geographic variability in MF incidence –highlighting over twice the incidence in metropolitan areas versus non‐metropolitan areas –supporting a potential role of environmental risk factors in MF pathogenesis.^24^

CBCLs accounted for 24.9% of PCLs in our study, which was only slightly higher than the approximately 24% reported in previous literature.^6^, ^12^ The most common CBCL subtypes were pcMZL, pcFL, and pcDLBCL.

This study is among the first in Germany to provide five‐year relative survival estimates for PCL subtypes in a population‐based setting. The overall five‐year relative survival for PCL was 89.0%, with notable differences by subtype: CTCL: 91.0%: MF: 96.2%, CD30+LPD: 98.2%, SS: 53.0%.

Our MF and CD30^+^LPD survival rates are consistent with findings from the Dutch and Austrian Cutaneous Lymphoma Groups (95% and 88%),^1^, ^25^, ^26^ and the Moroccan study (85.1%).^9^

Interestingly, our 53.0% survival rate in SS is markedly higher than historical estimates (20–42%) and closely aligns with a recent international multicenter study (53.4%), suggesting improved outcomes in recent years. This improvement may stem from the adoption of novel therapies such as brentuximab vedotin (anti‐CD30) and mogamulizumab (anti‐CCR4), both approved since 2018, as well as increasing use of combination therapies (e.g., extracorporeal photopheresis with retinoids or interferon).^27^, ^28^, ^29^

For CBCL, the five‐year relative survival was 86.0% with pcMZL: 92.3%, pcFL: 100%, pcDLBCL: 55.5%. Our pcMZL and pcFL survival estimates are comparable to previous European studies (99% and 95%).^1^, ^3^, ^9^ A slightly reduced relative survival rate in pcMZL may reflect inclusion of cases with secondary cutaneous involvement from systemic disease, as suggested in previous findings.^30^

Compared to international data, pcDLBCL patients in our registry showed higher survival (55.5%) than those in the French Study Group (41%) but lower than SEER data (64.7%). Reasons for this discrepancy remain unclear, particularly as treatment protocols are broadly harmonized between Europe and the U.S.^31^, ^32^, ^33^

Our data show an increasing incidence of CTCL and CBCL across both sexes over the 14‐year study period – mirroring trends seen in U.S. SEER data.^20^, ^21^, ^22^, ^23^, ^24^ In contrast, the incidence of other rare PCL types remained stable. These findings are largely consistent with our previous work based on statutory health insurance claims,^34^, ^35^ though notable differences exist in subtype distribution and trend detection, likely due to data quality and granularity limitations in claims‐based research.

The study covers also the years 2019–2021, overlapping with the COVID‐19 pandemic. Previous research has reported an increase in advanced‐stage MF/SS diagnoses during this period.^36^ However, due to the very low incidence of cutaneous lymphomas and the resulting large year‐to‐year fluctuations in incidence rates, it is virtually impossible to determine whether the pandemic or associated lockdowns led to a decline in diagnosis or care as shown for common cancers including melanoma and non‐melanoma skin cancers.^37^, ^38^, ^39^, ^40^ Furthermore, population‐based cancer registries – such as the one used in this study – often lack detailed clinical information on skin lymphomas, particularly staging data. As a result, potential shifts in stage distribution due to the pandemic cannot be meaningfully assessed.

A key limitation of this study is the lack of detailed clinical data (e.g., staging, treatment regimens, progression), which is currently being addressed by the ongoing implementation of population‐based clinical cancer registration. Linking clinical data with registry data will enhance data completeness and quality, enabling more nuanced analyses.

In conclusion, our study reveals *(1)* a rising incidence of PCL in Germany, with detailed insight into age‐specific patterns, including rare pediatric cases, *(2)* an increase in subtype‐specific diagnoses, particularly within CBCL, reflecting improvements in diagnostic precision over time, *(3)* for the first time in Germany, population‐based survival data for PCL, including improved outcomes in SS, likely due to advances in treatment.

These findings contribute valuable epidemiological evidence on PCL and support further development of targeted strategies in clinical practice and cancer control.

## CONFLICT OF INTEREST STATEMENT

C.A. has received advisory board fees from 4SC, Helsinn, Innate Pharma, Kyowa Kirin, Recordati Rare Diseases, and Takeda Pharmaceuticals. All other authors declare no conflicts of interest.

## Supporting information



Supplementary information

Supplementary information

Supplementary information
