# Supplementary figures and images for: Recent trends of incidence and mortality of cutaneous lymphomas in Germany
Source: J Dtsch Dermatol Ges. 2025 Dec 12;24(3):351–8. doi: 10.1111/ddg.15904 (PMC12968973; doi:10.1111/ddg.15904)

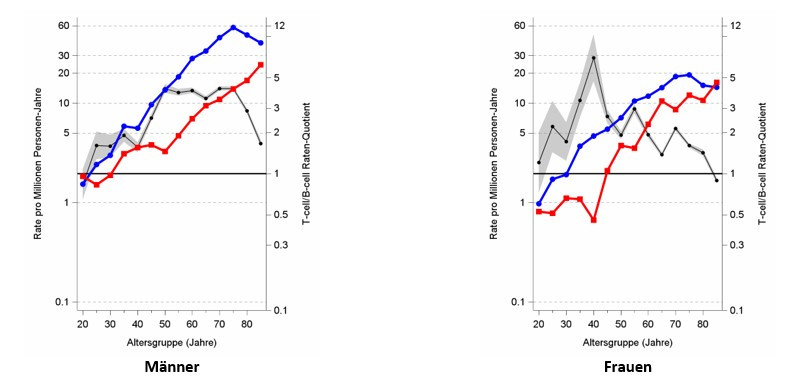

Supplement: Supplementary file 1 — Supplementary information [file DDG-24-351-s002.jpg]

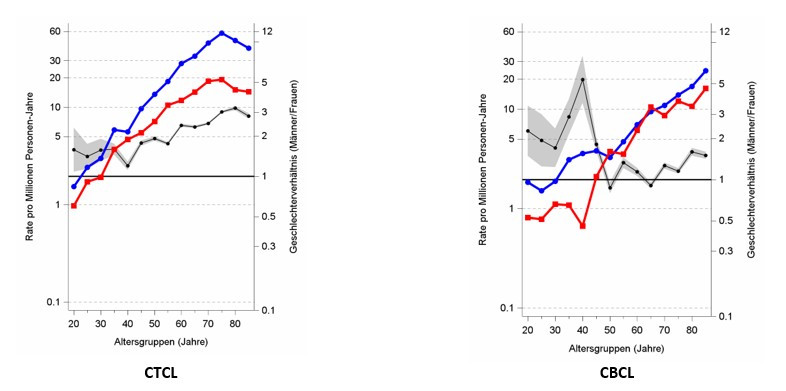

Supplement: Supplementary file 2 — Supplementary information [file DDG-24-351-s003.jpg]
