# Supplementary material for: Recent trends of incidence and mortality of cutaneous lymphomas in Germany
Source: J Dtsch Dermatol Ges. 2025 Dec 12;24(3):351–8. doi: 10.1111/ddg.15904 (PMC12968973; doi:10.1111/ddg.15904)
Supplement: Supplementary file 3 — Supplementary information [file DDG-24-351-s001.docx]

**Supplementary figures**

| **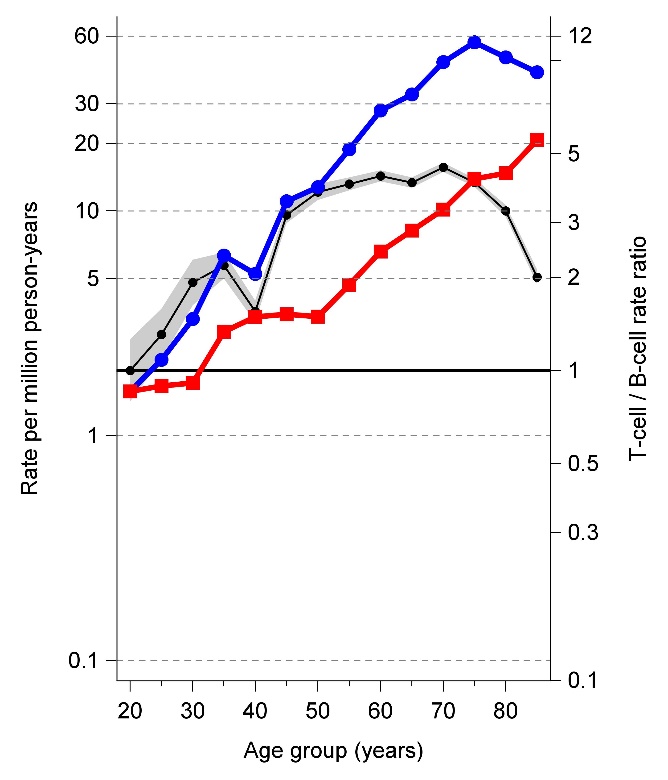** | **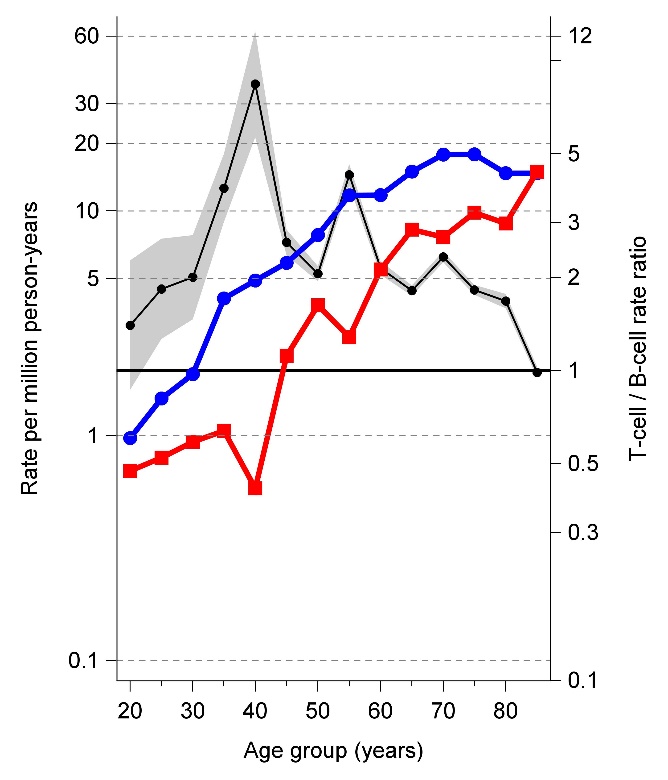** |
| --- | --- |
| Men | Women |

**Suppl. Figure 1: Ratio of age-specific incidence rates of cutaneous T-cell lymphomas and cutaneous B-cell lymphomas by sex in North Rhine-Westphalia, Germany, 2008-2021.** Incidence rate ratio of T-cell to B-cell lymphomas with 95% confidence intervals (grey bands). Blue graphs are cutaneous T-cell lymphomas, red graphs are cutaneous B-cell lymphomas black graph

| **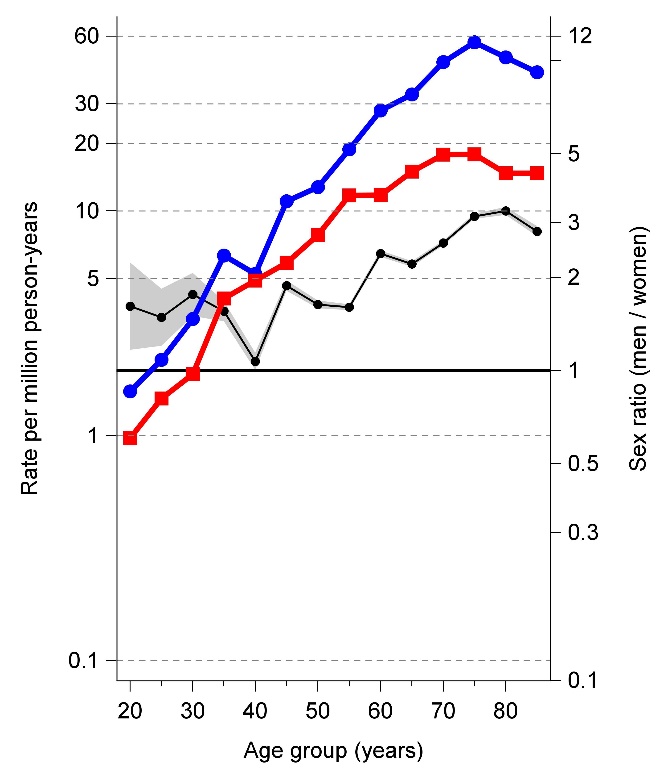** | **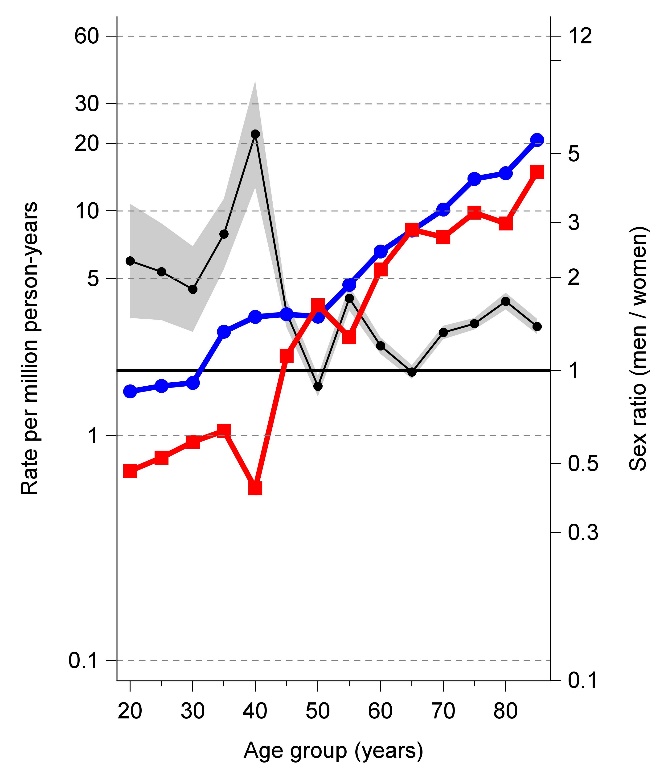** |
| --- | --- |
| CTCL | CBCL |

**Suppl. Figure 2**: **Sex-ratio of age-specific incidence rates of cutaneous T-cell lymphomas and cutaneous B-cell lymphomas in North Rhine-Westphalia, Germany, 2008-2021.** Blue graphs are men; red graphs are women; black graph: sex
